# Delirium risk factors in hospitalized patient: a comprehensive evaluation of underlying diseases and medications in different wards of a large Urban Hospital Center in Iran

**DOI:** 10.1186/s12871-022-01690-w

**Published:** 2022-05-16

**Authors:** Mohammad Arbabi, Elham Ziaei, Behnam Amini, Hamidreza Ghadimi, Fatemeh Rashidi, Narges Shohanizad, Soroush Moradi, Alireza Beikmarzehei, Alireza Hasanzadeh, Amirhossein Parsaei

**Affiliations:** 1 Brain & Spinal Cord Injury Research Centre, Tehran University of Medical Sciences, Tehran, Iran; 2 Psychosomatic Medicine Research Centre, Tehran University of Medical Sciences, Tehran, Iran; 3 Faculty of Medicine, Tehran University of Medical Science, Tehran, Iran; 4 Azad University of Medical Science, Tehran, Iran; 5 Non-communicable Diseases Research Center, Tehran University of Medical Sciences, Jalal Street, Tehran, 1411713139 Iran

**Keywords:** Delirium, Confusion, Hospitalization, Dementia, Neoplasms, Neurological diseases

## Abstract

**Background:**

Delirium is a neurobehavioral syndrome, which is characterized by a fluctuation of mental status, disorientation, confusion and inappropriate behavior, and it is prevalent among hospitalized patients. Recognizing modifiable risk factors of delirium is the key point for improving our preventive strategies and restraining its devastating consequences. This study aimed to identify and investigate various factors predisposing hospitalized patients to develop delirium, focusing mostly on underlying diseases and medications.

**Method:**

In a prospective, observational trial, we investigated 220 patients who had been admitted to the internal, emergency, surgery and hematology-oncology departments. We employed the Confusion Assessment Method (CAM) questionnaire, The Richmond Agitation Sedation Scale (RASS), the General Practitioner Assessment of Cognition (GPCOG), demographic questionnaire, patient interviews and medical records. Multivariate logistic regression models were used to analyze the predictive value of medications and underlying diseases for daily transition to delirium.; demographics were analyzed using univariate analysis to identify those independently associated with delirium.

**Results:**

Two hundred twenty patients were enrolled; the emergency department had the most incident delirium (31.3%), and the surgery section had the least (2.4%); delirium was significantly correlated with older ages and sleep disturbance. Among multiple underlying diseases and the medications evaluated in this study, we found that a history of dementia, neurological diseases and malignancies increases the odds of transition to delirium and the use of anticoagulants decreases the incident delirium.

**Conclusion:**

Approximately 1 out of 10 overall patients developed delirium; It is important to evaluate underlying diseases and medications more thoroughly in hospitalized patients to assess the risk of delirium.

## Introduction

Delirium is a neurobehavioral syndrome characterized by fluctuation of mental status, disorientation, confusion, and inappropriate behavior [1]. Disturbance of serum metabolites, neuroendocrine systems and neurotransmitters - especially cholinergic and dopaminergic systems- are assumed to play roles in delirium’s pathophysiology by disrupting the neuronal activity [2–4]. The point prevalence of delirium in patients over the age of 65 is more than 7.8% [5] and its incidence rate in hospitalized patients differs based on the underlying condition: it ranges from 5 to 87% [6, 7] -with overall higher incidences in intensive care unit (ICU) patients.

Delirium risk is determined by the interrelationship between predisposing factors (vulnerable background characteristics) and precipitating factors (acute insults or drugs). The total risk depends on each individual’s number of risk factors and severity. Delirium-related predisposing factors include increased age, cognitive impairment (such as dementia), comorbidities, psychiatric illness, and visual and hearing impairment [8, 9]. The precipitating factors vary depending on the settings and encompass a wide range of insults, including acute illness, surgery, dehydration and medications (use, interaction or withdrawal) [10]. Metabolic diseases such as hepatic encephalopathy, neuroendocrine disorders such as diabetes and certain classes of drugs like benzodiazepines, high dose narcotics, and anticholinergic medications have been linked to increased risk of delirium [11, 12]. Decreased perception of the environment caused by insufficient light and sleep disturbances increases delirium risk.

Delirium increases the mortality rate, lengthens hospitalization and places a heavy burden on hospitals and long-term care facilities [13]. Despite robust research on developing instruments to identify delirium, delirium remains underdiagnosed; A lack of accurate categorization of vulnerable patients has led to inadequate prevention guidelines for high-risk patients. So, to prevent delirium, its incidence rate in each medical section and its associated risk factors should be characterized properly. The purpose of our study is to provide means for the early diagnosis and prevention of this syndrome among hospitalized patients by identifying the possible risk factors; so in a prospective, observational cohort study of 220 patients of several medical sections, we determined the incidence of delirium and evaluated its associated risk factors.

## Methods

We enrolled patients from June to September 2019. All patients admitted to internal, surgical, emergency and hematology-oncology wards of Imam Khomeini hospital, Tehran, Iran, were evaluated, and a total of 220 patients were enrolled in the study via random sampling. Enrollment criteria included: 1- all patients 18 years or older, 2- not requiring mechanical ventilation. Exclusion criteria were as follows: 1- symptoms of withdrawal or intoxication (Based on clinical evaluations and Paraclinical tests and interviews with patient companions) 2- Decreased level of consciousness (RASS score − 4 or − 5, which considered as coma) [14, 15] 3- Patients receiving antipsychotics or high doses of morphine (> 60 mg/day) or midazolam (> 0.1 mg/kg/hr) or whom under general anesthesia and neuromuscular blocking agents recovering from surgery 4- Patients who were delirious at admission time 5- Inability to understand or speak Persian. Patients were followed up until they were discharged from the hospital or developed delirium.

At first, baseline demographics as well as information pertaining to known risk factors for delirium were obtained through patient interviews and medical records. These included age, sex, city of residency, level of education, marital status, employment status, number of family members and the patient’s general status information such as vision and hearing impairment and also access to visual and auditive aids if needed, underlying diseases and medications. We assessed sleep quality using The Pittsburgh Sleep Quality Index (PSQI) [16] and defined poor sleep quality as any score above 7 points [17–19]. The amount of sleep was evaluated based on the recommended amount of sleep per age range [20, 21], and if the amount of sleep was below the recommended range, it was considered insufficient sleep. Sleep habits were examined in order to identify alternate sleep patterns apart from night sleep.

**Fig. 1:**
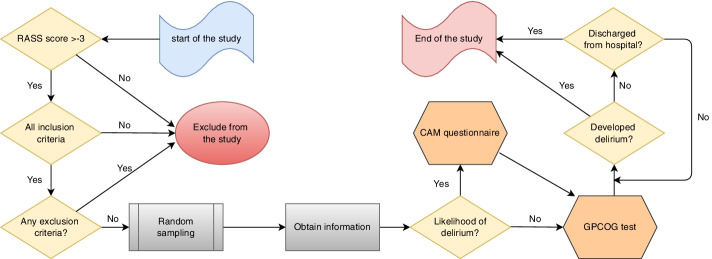
Flow chart illustrating the study design

As shown in Fig. 1, patients were evaluated once daily for delirium: at first, the level of consciousness was measured by The Richmond Agitation Sedation Scale (RASS) and - a RASS score of − 5 or − 4 is considered coma- if it exceeds − 4 (− 3 to + 4) then the confusion assessment method (CAM) questionnaire [22, 23] and the General Practitioner Assessment of Cognition (GPCOG) test was employed; GPCOG test was carried out on the first evaluation only, while RASS and CAM were performed on a daily basis until the patient was discharged or developed delirium. Initially, the researchers evaluated all patients thoroughly, and bedside nurses who had both RASS and CAM training conducted routine evaluations every day until the patients were discharged or developed delirium.

The Richmond Agitation Sedation Scale (RASS) is a valid instrument to measure the level of arousal; it is a 10-point scale ranging from − 5 to + 4: score of 0 represents a calm and alert state. Positive RASS scores indicate aggressive status and the negative RASS scores imply decreased levels of consciousness. The CAM questionnaire assesses the presence, severity, and fluctuation of 9 delirium features: acute onset, inattention, disorganized thinking, altered level of consciousness, disorientation, memory impairment, perceptual disturbances, psychomotor agitation or retardation, and altered sleep-wake cycle, and GPCOG test is used to evaluate patients for cognitive impairments and it is consisted of two components: a cognitive assessment and an informant questionnaire. The cognitive assessment includes nine items, each correct answer is valid one point leading to a maximum score of 9; results =9 or < 5 considered to be cognitively intact or impaired, respectively. Informant questionnaire only considered necessary if the cognitive test is equivocal (5-8 scores), the informant questionnaire queries an informant (person who knows the patient well) six historical questions, a score of 0-3 indicates cognitive impairment and requires further investigations.

### Statistical analysis

The Chi-square test investigated the correlation between nominal and ordinal variables. After that, the normality of variables was examined by Kolmogorov- Smirnov test; an independent t-test was performed to investigate the relationship between numeric variables with normal distribution and delirium. The Mann-Whitney U statistical test evaluated the difference between numeric variables with the abnormal distribution. Adjusted odds ratios (OR) and confidence intervals (CI) were calculated for factors with significant univariate correlations.

Logistic regression was used to evaluate the predictive value of the significant risk factors, and the Hosmer and Lemeshow test was used to assess the fit of the risk prediction models; delirium was considered as the dependent variable, and the significant risk factors as independent variables(x) and the risk factors were compared one by one with the dependent variable (delirium). Regression coefficients with 95%-confidence intervals (CI) and the corresponding *p*-values were calculated for each risk factor. Moreover, odds ratios (OR) with 95%-CI were determined in the logistic regression.

Since some patients had been concurrently afflicted by multiple diseases or been used multiple drugs, with regards to confounding effects of underlying diseases or drugs on each other, justified odds ratios were calculated. However, the adjusted odds ratios were not calculated in these two subgroups due to the low number of patients with hyperlipidemia and liver disorders.

All data were analyzed using the 11th version of Stata, and the first-order error(α) equal to 0.05 was considered statistically significant.

## Results

Of the 220 patients studied, 114 were male (51.9%), and the average age was 59.3 years (SD = 13). Of these patients, 112 patients (50.9%) were hospitalized in the internal ward, 83(37.7%) in the surgery section, 16(7.2%) in the emergency department and 9(4.09%) in the hematology-oncology ward. The overall incidence of delirium was 10%, and we obtained the highest incidence in the emergency department (31.3%) and the least in the surgical section (2.4%). Patients developed delirium on average 4 days after hospitalization, ranging from 1 to 14 days (median = 2, IQR = 3).

The overall incidence of delirium in the study was 10%, which differed in various sections of the hospital; we obtained the incidence of delirium as follows: emergency department 31.3%, hematology-oncology ward 22.2%, internal ward 11.6%, and surgery section 2.4% (Table 1).

**Table 1:** Incidence of delirium by ward

Ward	*Affected*	Not affected	Incidence
Internal	13	99	11.6%
Surgery	2	81	2.4%
Emergency	5	11	31.3%
Hematology-Oncology	2	7	22.2%
**Total**	**22**	**220**	**10%**

By analyzing demographic indicators, we found significant association between age (*p*-value = 0.014) and the incidence of delirium; Among delirious patients, the average age was 65.7, while it was 58.6 among those who were not delirious. However, there was no significant correlation between incidence of delirium and gender (*p*-value = 0.857), education (*p*-value = 0.414), city of residency (*p*-value = 0.386), employment status (*p*-value = 0.395), living with family members (*p*-value = 0.178) and marital status (*p*-value = 0.093).

We found that the incidence of delirium was significantly associated with dementia (*p*-value = 0.008) and had a negative correlation with the quantity of sleep hours (*p*-value < 0.0001) and its quality (*p*-value < 0.0001). However, there was no significant correlation between type of vision and hearing impairment (*p*-value = 0.336, 1.000 respectively), use and access to hearing aids (*p*-value = 1.000, 1.000 respectively) and visual aids (*p*-value = 0.052, 0.274 respectively), sleep habits (*p*-value = 1.000), and visual and hearing health status (*p*-value = 0.056, 0.795 respectively) (Table 2).

**Table 2:** Demographics and delirium interview information of the patients

Factors	Delirium		*P*-value
	Affected No. (%)	Not-affected No. (%)	
**Vision**			
Intact	3(13.64)	67(34)	0.056
Impaired or blind	19(86.36)	130(66)	
**Type of visual loss**			
Partial	18(94.7)	128(98.5)	0.336
Total	1(5.3)	2(1.5)	
**Use of eyeglasses**			
Yes	6(33.3)	75(57.7)	0.052
No	13(66.7)	55(42.3)	
**Access to eyeglasses**			
Yes	3(16.7)	41(31.5)	0.274
No	15(83.3)	89(68.5)	
**Hearing**			
Intact	17(77.3)	148(74.7)	0.795
Impaired or deaf	5(22.7)	50(25.3)	
**Type of hearing loss**			
Partial	5(100)	47(94.2)	1.000
Total	0(0)	3(5.8)	
**Use of hearing aid**			
Yes	0(0)	5(9.8)	1.000
No	5(100)	45(90.2)	
**Access to hearing aid**			
Yes	0(0)	3(5.9)	1.000
No	5(100)	47(94.1)	
**Dementia**			
Yes	3(13.6)	2(1)	0.008
No	19(86.4)	196(99)	
**Comfortable sleep**			
Yes	2(9.1)	122(61.6)	0.0001
No	20(90.9)	76(38.4)	
**Adequate sleep**			
Yes	3(13.6)	171(86.3)	0.0001
No	19(86.4)	27(13.6)	
**Sleep habits**			
Yes	1(4.5)	15(7.6)	1.000
**No**	**21(95.5)**	**183(92.4)**	
**Sex**			
Male	11 (50)	103 (52)	0.857
Female	11 (50)	95 (48)	
**City of residence**			
Capital	11 (50)	80 (40.4)	0.386
Others	11 (50)	118 (59.6)	
**Education**			
Illiterate	11 (50)	75 (37.9)	0.414
Under diploma	9 (40.9)	81 (40.9)	
Diploma	0 (0)	21 (10.6)	
University degree	2 (9.1)	21 (10.6)	
**Marital status**			
Married	13 (59.1)	152 (76.8)	0.093
Single	0 (0)	5 (2.5)	
Widow	9 (40.9)	35 (17.7)	
Divorced	0 (0)	6 (3)	
**Employment status**			
Employed	3 (13.6)	37(18.7)	0.395
Retired	2 (9.1)	38 (19.2)	
Unemployed	17 (77.3)	123 (62.1)	
**Insurance**			
Yes	12 (54.6)	122 (61.6)	0.519
No	10 (45.4)	76 (38.4)	
**Living with family members**			
Yes	19 (86.4)	186 (93.9)	0.178
No	3 (13.6)	12 (6.1)	
**Age**	65.7^a^(12.8^b^)	58.6(12.8^b^)	0.014

^a^mean age of the group

^b^standard deviation

Since some patients had several diseases simultaneously, adjusted statistical ratios were used to eliminate this confounding factor and based on that, we found a significant association between delirium and underlying dementia, neurological diseases and malignancies. Nevertheless, we did not find any significant correlation between delirium and diabetes, hypertension, hyperlipidemia, cardiovascular disease, renal, hepatic, pulmonary and infectious diseases (Table 3).

**Table 3:** Analyzing the underlying diseases and medications of the patients for delirium

Underlying disease	adjusted		Drug group	adjusted	
	*O*R *(CI)*	*P-value*		*O*R *(CI)*	*P-value*
**Dementia**	10.6(1.2-93.9)	0.034	**Anti-diabetics**	*0.5(0.1-2.3)*	*0.152*
**Diabetes**	2.6(6.9-7.0)	0.072	**Anti-hypertensive**	0.7(0.1-3.7)	0.432
**Hypertension**	1.1(3.0-5.3)	0.843	**Diuretics**	0.4(0.1-1.3)	0.124
**Hyperlipidemia**	–	–	**Antibiotics**	2.3(0.5-9.8)	0.086
**Cardiovascular disease**	0.9(1.2-3.0)	0.874	**Anti-hyperlipidemics**	0.6(0.1-2.8)	0.361
**Kidney disease**	1.1(2.2-6.0)	0.951	**Analgesics**	1.4(0.3-5.6)	0.463
**Liver disease**	–	–	**Anti-coagulants**	0.3(0.0-1.9)	0.029
**Neurologic disease**	8.1(6.1-31.2)	0.002	**Chemotherapy**	0.2(0.1-3.2)	0.078
**Malignancies**	3.3(8.0-10.1)	0.048	**Anti-convulsant**	0.2(0.1-3.2)	0.078
**Lung disease**	1.4(7.2-12.0)	0.737	**Sedatives**	2.1(0.16-3.5)	0.481
**Infectious disease**	1.2(5.9-15.0)	0.899	**Opioids**	0.9	0.861

We found a significant correlation between anticoagulant use and delirium. No significant association was found between delirium and use of anti-diabetic, anti-hypertensive, diuretics, antibiotics, anti-hyperlipidemics, analgesics, chemotherapy agents, sedatives, opioids and anticonvulsants; nevertheless, antibiotics(*p*-value = 0.086), chemotherapy agents(*p*-value = 0.078) and anticonvulsants (*p*-value = 0.078) had a near significant *p*-value and needed to be further investigated.

## Discussion

Our study is a prospective, observational trial that evaluated the incidence rate of delirium and its contributing risk factors in 220 patients admitted to the internal, emergency, surgery and hematology-oncology departments.; first, we found that 1 out of 10 overall patients developed delirium; the emergency department had the most incident delirium (31.3%), and the surgery section had the least (2.4%). Second, we found a meaningful positive correlation between the incident delirium and older ages and sleep disturbance by assessing the demographic indicators and general status information of included patients. However, we found no significant correlation between delirium and gender, employment status, sleep habits, living with family members, marital status, educational degree, visual or auditory impairment and access to visual and hearing aids. Third, by utilizing multivariate regression analysis for multiple underlying diseases and medications, we found that history of dementia, neurological diseases and malignancies increases the odds of transition to delirium and the use of anticoagulants decreases the incident delirium. However, the correlation of delirium and other underlying diseases like diabetes, hypertension, cardiovascular disease, renal, pulmonary and infectious diseases, and other medications was not meaningful.

The 10% incidence of delirium in this study is consistent with previous reports [24] and is lower than the higher incidences reported by most of the other studies [24, 25]. This salient discrepancy might be originated in differences in patient characteristics (e. g., the average age, the severity of underlying condition, type of the diseases), the screening instrument and its application and considering the drug-induced sedation and medication-induced coma as delirium. Although the use of visual aids and the state of vision did not significantly predict the development of incident delirium, their significance levels (*p*-values: 0.56 and 0.52 respectively) were marginal, and both increased the odds of developing delirium. There was no association between hearing status or use of hearing aids and delirium risk in our study, but due to the small number of patients with hearing impairment in our study, the generalizability of these data is doubtful due to low statistical power. Furthermore, the observed significant differences in delirium incidence among the wards under-study could be due to differences in follow-up length, the patient characteristics and their specific medications [26]; the lower incidence of delirium in the surgical ward compared to the internal ward could be due to younger ages and also, most of them were at non-urgent surgery condition with a good health background.

As reported by previous studies, sleep hours and its quality have a strong correlation with delirium. The disturbed neurotransmission underlies this relationship: the REM cycles of the sleep adjust the acetylcholine and dopamine neurotransmission, and both cholinergic and dopaminergic systems are reported to be dysregulated in the delirium state [27, 28]. Sleep habits, like sleep time or afternoon naps, have not been associated with incident delirium. Baseline cognitive deficits were associated with an increased risk of developing delirium [2]; dementia and neurological disorders, through decreasing cerebral oxidative metabolism, cholinergic deficiency and inflammation increase the odds of developing delirium [29].

Malignancies increase the chances of developing delirium through their adverse effects on the immune system, the blood-brain barrier, and the nervous system [30]. The number of patients with malignancy involved in this study was not enough to evaluate the specific subtypes of malignancies for delirium, but according to previous studies, the patients with primary or secondary CNS tumors, cancers with paraneoplastic neurological features and terminal cancers are at higher risks to develop delirium [31, 32]; however, taking the chemotherapy medications that were reported as a predisposing factor for delirium in previous studies, were not associated with higher risks of delirium in our study. Because of the insufficient number of patients with hyperlipidemia and liver disease, we were not able to analyze these two variables; according to previous studies, hyperlipidemia plays a protective role by strengthening the blood-brain barrier, and liver diseases by disturbing plasma metabolites and electrolytes are risk factors for delirium [33–35].

Despite other studies reporting a relationship between delirium and diabetes [36], we could not find an association between them; diabetes mellitus is a chronic disease that is often accompanied by other diseases and also, diabetic patients receive numerous medications; hence, to find out whether diabetes increase the odds of developing delirium, these all covariates should be evaluated cautiously. Siew et al. [37] reported that acute kidney injury increases the odds of developing delirium, but we only evaluated the chronic kidney diseases in this study and did not find a meaningful relationship. Infectious diseases like urinary tract infections were reported to increase the chance of delirium [38], but infectious diseases were not associated with delirium in our study due to the lack of categorization and younger ages of our participants. The lack of relationship between delirium and cardiac and pulmonary disorders, which we obtained, resembles previous studies [33].

Previous studies’ assessments of the role of medications in developing delirium are limited and inconsistent [39, 40]. So, we investigated the role of a wide range of prescribed drugs for developing delirium. Anticoagulants (warfarin, heparin, enoxaparin) reduced the chances of developing delirium, similar to a previous study by Diez-Manglano et al. [41] which found anticoagulants to be associated with lower delirium prevalence in patients with atrial fibrillation. Nevertheless, Lahariya et al. [42] reported an increase in delirium risk by receiving warfarin in patients admitted to a cardiac intensive care unit, while Ranhoff et al. [43] found no relation between warfarin use and the risk of delirium. However, we could not find any relationship between delirium and other drugs, but the significance levels of chemotherapy agents and antibiotics were marginal, and both increased the odds of developing delirium.

Several limitations of this investigation warrant consideration: first, our sample size limited our ability to evaluate some underlying diseases like liver diseases and hyperlipidemia and specific subtypes of diseases like malignancies or drugs like chemotherapy medications and antibiotics. Second, our study did not consider any laboratory values because the diagnostic laboratory values for delirium are still in advance [44–46]. Third, we evaluated delirium once daily; based on the fluctuating nature of delirium, some cases may have been missed; by assessing patient’s cognitive status more frequently (every 4–8 hours), this bias would be removed, but this task is difficult to accomplish in a research setting due to resource and time constraints and also it is burdensome to patients. Fourth, we investigated numerous covariates deemed relevant a priori; so, other covariates that were not measured might have affected our results. The strengths of this study lay in its diverse sample of medical patients with different types of conditions.

## Conclusion

In summary, we investigated a wide range of medical and demographical factors to find the predisposing and precipitating factors of delirium; this study documents delirium’s incidence and risk factors in a prospective study of patients admitted in different sections of a referral hospital. The adverse outcomes of developing delirium are burdensome for the healthcare system and are accompanied by the decreased quality of life and increased mortality and morbidity of hospitalized patients. Therefore, recognizing the predisposing factors of delirium is the first step to preparing the healthcare systems to decrease the incidence and restrain the consequences. Future studies would need to explore that by which molecular and biological mechanisms, the known risk factors of delirium increase its occurrence and also, more interventional studies in this area are needed to strengthen our preventive and therapeutic strategies that, at the moment, are not effective enough to prevent or cure delirium in most of the patients.

## Data Availability

Researchers can obtain the datasets used in the current work from the Corresponding author upon request. The data sharing policies in the Tehran University of Medical Sciences follow local regulations of our institution.
